# Gastric Carcinoid: The Invisible Tumor!

**DOI:** 10.7759/cureus.13556

**Published:** 2021-02-25

**Authors:** Samyak Dhruv, Shamsuddin Anwar, Abhishek Polavarapu, Deeb Liliane

**Affiliations:** 1 Internal Medicine, Northwell Health, New York, USA; 2 Gastroenterology, Northwell Health, New York, USA; 3 Gatsroenterology, Northwell Health, New York, USA

**Keywords:** gastric tumor, gastrointestinal carcinoid tumor, upper endoscopy

## Abstract

Neuroendocrine tumor (NET) of the stomach or gastric carcinoid (GC) is a rare tumor derived from enterochromaffin-like (ECL) cells of the stomach and is more common in women after the fifth decade of life. The incidence of GC has been recently trending up. While most GC are visible lesions upon direct visualization on endoscopy, one-fourth of these tumors are intramucosal and not readily identified on upper endoscopy. Thus, a complete gastric map with biopsies of antrum, body, and fundus is required to confirm the presence of carcinoid growth. Herein we report a rare case of GC which was identified on a random gastric biopsy specimen.

## Introduction

Neuroendocrine tumors (NETs) are a highly heterogeneous group of neoplasms that originates from the cells of the endocrine and nervous systems distributed throughout the body. They most commonly arise from the gastrointestinal tract where they are called carcinoids [1]. Such tumors arising from the gastric mucosa are defined as gastric NET or gastric carcinoid (GC). These neoplasms are rare comprising 1% of all NETs and 1.8% of gastric cancers [2]. The incidence of GC is around 4.85 per 1,00,000 patients but has been recently trending up [3]. This is being attributed to the wider availability of endoscopies and extensive use of acid-suppressive medications leading to secondary hypergastrinemia, enterochromaffin-like (ECL) cell hyperplasia, dysplasia, and ultimately neoplasia [4]. They usually have a benign course with few exceptions. We intend to describe an interesting case of a young female diagnosed with type 1 gastric NET incidentally discovered on the random biopsy specimen.

This case report was previously presented as an abstract. (Abstract: Dhruv S, Anwar S, Polavarapu A, Deeb L. S3578 Gastric Carcinoid - The Invisible Tumor!!, The American Journal of Gastroenterology; October 2020) https://journals.lww.com/ajg/Fulltext/2020/10001/S3578_Gastric_Carcinoid___The_Invisible_Tumor__.3576.aspx

## Case presentation

**Figure 1:**
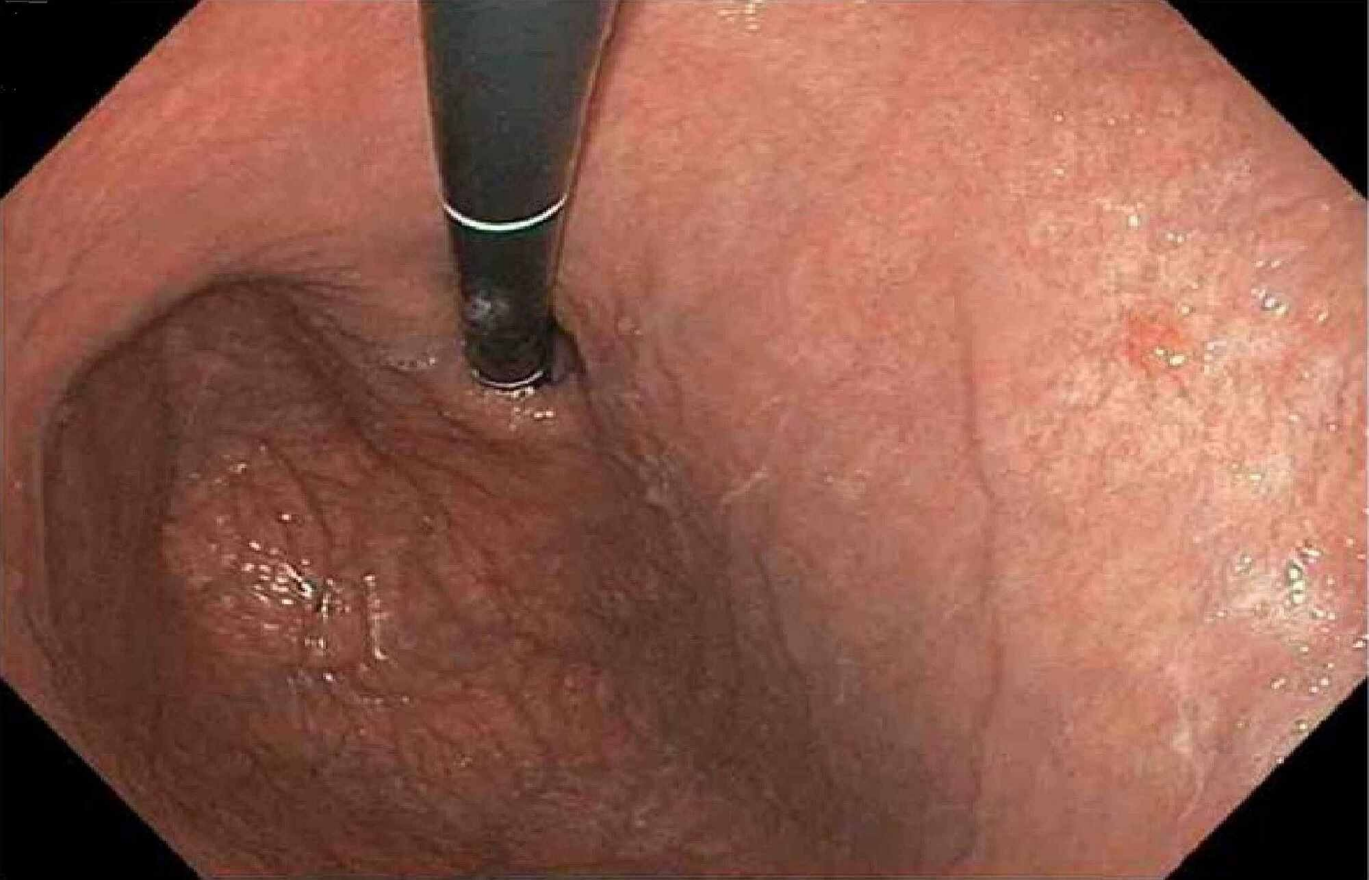
Retroflexed endoscopic view of gastric fundus and corpus showing flattened gastric folds with prominent, visible submucosal vessels consistent with chronic atrophic gastritis.

**Figure 2:**
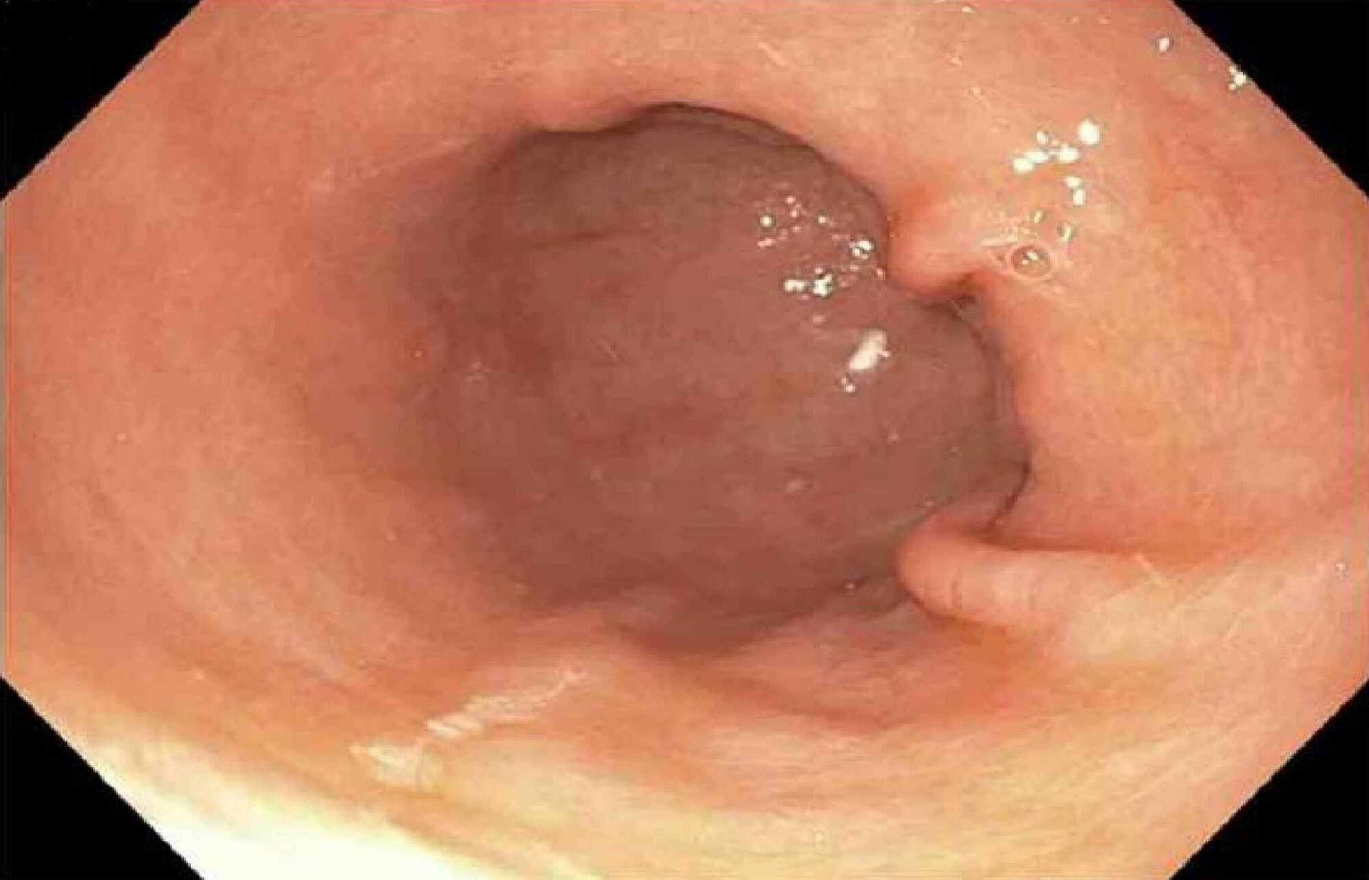
Endoscopic image of gastric corpus and antrum showing flattened gastric folds with pale mucosa.

A 45-year-old female with autoimmune pernicious anemia and Hashimoto's thyroiditis was being monitored by upper endoscopy to screen for type 1 GC and gastric adenocarcinoma in the setting of autoimmune chronic atrophic gastritis (CAG). She initially underwent her first upper endoscopy in 2015 for anemia workup which showed flattened gastric folds and 'no visible lesions' (Figures 1-2). Random gastric biopsies disclosed a 2.5 mm NET involving the lamina propria of the stomach body in the background of autoimmune CAG. The Ki-67 <2% confirmed it as grade 1 well-differentiated type 1 NET of the stomach. Immunohistochemical staining was positive for chromogranin A and synaptophysin and negative for cytokeratin 7 and 20, which was in favor of carcinoid tumor. Annual surveillance endoscopies were performed after initial diagnosis but they failed to show any gross lesions, atrophic changes in fundus or body, and repeated random gastric biopsies remained negative for carcinoid recurrence till 2019. She did not follow up after that.

## Discussion

NETs are epithelial cell neoplasms with characteristic neuroendocrine cells that may arise throughout the body but are usually present in the mucosa of the pancreas or the gastrointestinal tract. All NETs are graded into three categories (Grade 1, 2, and 3) according to the Ki-67 mitotic index which utilizes special staining to count the number of cells undergoing mitotic changes. Generally, the higher Ki-67 mitotic index corresponds to poor differentiation of the neoplastic cells with few exceptions [1]. Along with the grading, the staging (tumor‐node‐metastasis (TNM) system) of the neoplastic process is determined by biopsy and imaging studies such as computed tomography (CT) scan, magnetic resonance imaging (MRI), or octreotide scintigraphy [5]. Immunohistochemical analysis of the biopsy specimen is essential in the diagnosis of NETs. Chromogranin A and synaptophysin are currently considered the most specific immunohistochemical markers for NETs [6].

Functionally, NET of the stomach originates from ECL cells of mucosa and comprise approximately 1.8% of all the gastric malignancies [7]. In the United States, from 2000 to 2012, Surveillance, Epidemiology and End Results Program (SEER) 18 Registry data indicated that only 7%-8% of gastrointestinal NETs are found in the stomach [8]. Persistent anemia as discussed in our patient is considered to be the most common reason for evaluation of a patient with an upper endoscopy leading to incidental detection of NETs in the stomach. Some patients with GC have acid reflux/gastroesophageal reflux disease (GERD) or gastrointestinal bleeding leading to their endoscopic evaluation and eventual diagnosis of GC [9].

The ECL cells in normal individuals synthesize and secrete histamine in response to stimulation by hormone gastrin which itself is secreted by gastric epithelial G cells. The primary function of histamine and gastrin is positive feedback to parietal cells to enhance acid secretion. The NETs of the stomach are categorized into three subgroups depending upon their immunohistochemical and prognostic features [10].

Type 1 GC is associated with CAG. Gastrin level rises in response to chronic achlorhydria resulting in hyperplasia of neuroendocrine cells followed by dysplasia and potential development of NETs [11]. The annual incidence of type 1 GC in patients with CAG is around 0.4% [12]. Symptoms are vague abdominal pain and iron deficiency anemia. In 22.2% of type 1 GC, tumors are microscopic ranging in size from 0.5 to 5 mm, typically discovered incidentally on random gastric biopsies as in our patient, hence the name “invisible tumor”. Subcentimetric lesions can be resected endoscopically. Recurrence after resection may reach up to 65% on first year follow up, highlighting the importance of surveillance. The National Comprehensive Cancer Network (NCCN) guidelines recommend esophagogastroduodenoscopy (EGD) yearly after resection of GC for the first three years. However, an optimal follow up schedule is yet to be established [13].

In type 2 GC excessive gastrin is being released from a secondary focus such as from gastrinoma in pancreas or duodenum (Zollinger-Ellison syndrome) stimulating multiple foci of gastric NETs. After confirming the diagnosis of gastric NET, focus should be placed at localizing the gastrinoma and its surgical resection if possible.

Unlike type 1 and 2 tumors, type 3 comprises of variety of endocrine cells and may be associated with atypical carcinoid syndrome. The serum gastrin levels are usually normal in these patients. Given its aggressive nature, locally invasive or metastatic disease is often present at the time of diagnosis and recommended management includes gastric resection (partial vs total) along with local lymph nodes involved. Some studies suggest, in the absence of lymphatic invasion, localized disease to the lamina propria and submucosa may be treated with endoscopic removal, however the standard treatment remains to be resection [14]. In unresectable disease, local therapies such as arterial embolization, chemotherapy and radio ablation can be utilized with aim of reducing the progression of disease and improve survival. Table 1 below summarizes some of the key features of different types of NETs of the stomach.

**Table 1:** Table summarizing some of the key features of different types of neuroendocrine tumors (NETs) of the stomach. [15] EGD: esophagogastroduodenoscopy; MEN 1: multiple endocrine neoplasia type 1.

FEATURES	TYPE 1	TYPE 2	TYPE 3
Frequency (% among gastric NETs)	70-80%	5%	20%
Associations	Atrophic gastritis, Pernicious anemia	Zollinger-Ellison Syndrome, MEN 1	None
Characteristics	Multiple, small polypoid lesions in fundus	Multiple, small polypoid lesions in fundus	Large, solitary lesion in fundus/body
Clinical Behavior	Indolent	Indolent	Aggressive
Gastrin Levels	Elevated	Elevated	Normal
Management	<2 cm-Endoscopic Resection >2 cm-Surgical Resection	<2 cm-Endoscopic Resection >2 cm-Surgical Resection	Gastrectomy with lymph node resection
Surveillance post treatment	EGD every 6-12 months	EGD every 6-12 months	Imaging every 6 months after first year of surgery then annually for 10 years after surgery

In few studies, a rare form of NET of the stomach called type 4 GC has also been described as multiple small hypertrophic and hyperplastic parietal cell lesions with vacuolated cytoplasm. The main abnormality is the inability of the neoplastic parietal cells to secrete hydrochloric acid (HCl) causing achlorhydria, hypergastrinemia and ultimately triggering the proliferation of neuroendocrine cells [13].

After resection of well differentiated NET, there is limited evidence available for recommendations in terms of surveillance for recurrence. Long term surveillance with imaging studies including MRI and CT scans is strongly suggested for gastric NET types 1 and 2 that are greater than 2 cm in size, as relapse in distant places may occur beyond five years after surgery. The NCCN suggests EGD every one to two years in gastric NETs <2 cm in size [16]. As NET type 3 is considered to be similar in behavior as gastric adenocarcinoma, chemotherapy and radiation can be added as therapeutic options after radical resection.

In general, type 1 and 2 gastric NETs have good prognosis with close surveillance. Type 3 may have high five-year mortality rate (75%-87%) in poorly differentiated tumors. Type 4 is considered to have the worst prognosis with a mean survival of 6.5-14 months after diagnosis with a mortality rate of 100% in five years [17].

The role of biomarkers such as chromogranin A and 5-hyrdoxyindoleacetic acid (5-HIAA) in monitoring of disease recurrence has been debatable in medical literature and is currently not recommended as a standard strategy for routine surveillance [18].

## Conclusions

Gastric NETs are broadly differentiated into three types and all of them have a different therapeutic and surveillance strategies. So, appropriate diagnosis via endoscopy and histopathological classification are important for management decisions. Although rare, it is essential to keep GC in the differential diagnosis of gastric malignancies. This article also highlights the need for having evidence-based surveillance strategies for gastric NET which is still lacking at this point, given the rarity of this disease.
